# Upfront metastasis-directed therapy in oligorecurrent prostate cancer does not decrease the time from initiation of androgen deprivation therapy to castration resistance

**DOI:** 10.1007/s12032-021-01518-6

**Published:** 2021-05-18

**Authors:** Luca Triggiani, Rosario Mazzola, Davide Tomasini, Alessio Bruni, Giulia Alicino, Fabio Matrone, Roberto Bortolus, Giulio Francolini, Beatrice Detti, Alessandro Magli, Marco Lorenzo Bonù, Gianluca Ingrosso, Andrea Lancia, Fabio Trippa, Ernesto Maranzano, Ciro Franzese, Paolo Ghirardelli, Vittorio Vavassori, Marta Scorsetti, Filippo Alongi, Stefano Maria Magrini

**Affiliations:** 1 Department of Radiation Oncology, University and Spedali Civili Hospital, Piazzale Spedali Civili 1, 25123 Brescia, Italy; 2 Department of Advanced Radiation Oncology, IRCCS Sacro Cuore Don Calabria Hospital, Negrar Di Valpolicella, Verona, Italy; 3 Department of Oncology and Hematology, Radiotherapy Unit, University Hospital of Modena, Modena, Italy; 4 Department of Radiation Oncology, Centro Di Riferimento, Oncologico Di Aviano CRO-IRCCS, Aviano, Italy; 5 Department of Radiation Oncology, University of Florence, A.O.U Careggi, Florence, Italy; 6 Department of Radiation Oncology, University Hospital of Udine, ASUIUD, Udine, Italy; 7 Radioterapia Oncologica, Dipartimento di Scienze Chirurgiche E Biomediche, Università degli Studi di Perugia, Ospedale S. Maria della Misericordia, Perugia, Italy; 8 Radiation Oncology, Fondazione IRCCS Policlinico S. Matteo, Pavia, Italy; 9 Department of Radiation Oncology, ‘S. Maria’ Hospital, Terni, Italy; 10 IRCCS, Radiotherapy and Radiosurgery Department, Humanitas University Hospital, Milan-Rozzano, Italy; 11 Cliniche Gavazzeni-Humanitas, Radiotherapy, Bergamo, Italy

**Keywords:** Prostate cancer, Metastasis directed therapy, Stereotactic body radiotherapy

## Abstract

The aim of the present study was to explore the potential impact of upfront metastases-directed therapy (MDT) in terms of prolongation of castration-sensitive phase in a series of oligorecurrent castration-sensitive prostate cancer (PC) patients. The present article is a multicenter retrospective study. The population of interest was castrate-sensitive oligorecurrent PC, defined as the presence of 1–3 uptakes in non-visceral sites such as bones or nodes detected by means of 18F-Choline PET/CT or 68-Gallium PSMA PET/CT. Primary endpoint was the time to castration resistance. Secondary endpoints were ADT-free survival, local progression-free survival, and overall survival. Eighty-two patients and 118 lesions were analyzed. The median time to castration resistance for the entire population of the study was 49 months (95% CI 43.6–54.4 months). The 1- and 2-year TTCR-free survival rates were 94% and 82%, respectively. At the time of analysis, 52 patients were still in the castration-sensitive phase of the disease. In this cohort of patients, the median ADT-free survival was 20 months (range 3–69 months). On the other hand, during follow-up 30 patients switched to the castration-resistant phase of disease. In this last group of patients, the median ADT-free survival was 20 months (range 4–50 months). After the ADT administration, the median castration-sensitive phase was 29 months (range 5–71 months). Castration resistance generally occurs at a median follow-up of 24–36 months following ADT. In the current study, upfront MDT does not decrease the time from initiation of ADT to castration resistance.

## Introduction

Historically, the management of metastatic castration-sensitive prostate cancer (PC) relied on the administration of androgen deprivation therapy (ADT) with Luteinizing Hormone-Releasing Hormone (LH-RH) analogues or antagonist. In this clinical setting, ADT is able to prolong the biochemical control being able to impact on survival outcomes [1]. Unfortunately, ADT long-term effectiveness is limited, even if initial response with a fast PSA decrease is quite common after the first LHRH analogues administration. Indeed, after first-line ADT, disease progression represents a common event due to the development of the castration--resistant phase that generally occurs at a median follow-up of 24–36 months [2–4]. For this reason, ADT for metastatic PC is largely considered a palliative treatment and, in addition, the administration of ADT itself can be burdened by the onset of non-negligible adverse events such as cardiovascular events, metabolic syndrome, sarcopenia, sexual dysfunction, and osteoporosis [5]. These issues have to be particularly taken into account due to the long-term duration of this first line option and the relevance it may have particularly in frail or older patients. In the setting of oligorecurrent castration-sensitive PC a substantial amount of literature experiences, characterized by several levels of evidence, recently showed that metastasis-directed therapy (MDT) using high dose radiotherapy (SBRT) might represent a viable curative option able to improve disease control and significantly delay the administration of palliative ADT [6–8]. It is currently object of debate if MDT is able to prolong the time to castration resistance (TTCR) onset or negatively modify the natural history of the castration-sensitive PC.

The main aim of the present study was to explore the potential impact of upfront MDT in terms of prolongation of castration-sensitive phase in a series of oligorecurrent castration-sensitive PC patients.

## Methods

The present article is a multicenter retrospective study. The inclusion criteria were (i) histologically confirmed diagnosis of acinar adenocarcinoma of the prostate, (ii) biochemical relapse after primary tumor treatment (radical prostatectomy or radical RT) defined according to European Association of Urology guidelines and/or according to Phoenix criteria in post-surgical or post-RT setting respectively [1], (iii) castrate-sensitive oligorecurrent PC, defined as the presence of 1–3 uptakes in non-visceral sites such as bones or nodes detected by means of 18F-Choline PET/CT (Cho-PET) or 68-Gallium PSMA PET/CT (PSMA PET), (iv) controlled primary tumor, (v) patients treated with upfront MDT for oligorecurrences without ADT, (vi) SBRT directed to every site of disease and delivered with ≥ 5 Gy dose per fraction.

### SBRT and follow-up

Before SBRT, all patients underwent a CT-based treatment planning with 1–3 mm slice thickness in the supine position. Gross tumor volume (GTV), equal to clinical target volume (CTV), was delineated using all the available morphological and metabolic imaging information. A planning target volume (PTV) was created around the CTV using isotropic or anisotropic 3–5 mm margins. Organs at risk were then delineated depending on the location of the target volume.

SBRT schedules followed the local treatment policy of each center varying between 25 and 50 Gy in 3 to 7 fractions. Image-guided intensity-modulated RT with static beams or dynamic arcs were used, depending on each center internal protocols and technology availability. At each fraction, the patient’s set-up and target accuracy was always verified using on-board cone-beam or megavolt CT.

After SBRT, all patients were followed every 3–4 months with clinical evaluation and PSA blood tests. Cho-PET or PSMA PET scans were performed within 3 months after SBRT and every 3 months thereafter in the case of detectable PSA or biochemical progression post-SBRT, defined according to PC Working Group 3 [9]. Instrumental tumor response was assessed according to the PET Response Criteria in Solid Tumors [10].

If oligo-progression occurred after MDT, further courses of SBRT were proposed if less than 3 new lesions were detected by molecular imaging outside the previously irradiated field. Finally, ADT was administered when the conversion in the polymetastatic castration-sensitive phase of the disease occurred (more than three new metastases onset).

### Study endpoints and statistical analysis

The primary endpoint of the current study was the TTCR defined according to EAU guidelines [1].

Secondary endpoints were ADT-free survival, local progression-free survival (LPFS), overall survival (OS). Detailed SBRT-related adverse events were also reported. TTCR was defined as the time between the starting of ADT after SBRT and three consecutive rising in PSA resulting in two 50% increases over the nadir and PSA > 2 ng/ml (castrate serum testosterone < 50 ng/dl).

ADT-free survival was defined as the time between the last session of SBRT and the administration of ADT for the polymetastatic disease conversion. The LPFS was defined as the time between the last session of SBRT and the progression of the disease within the target volume. OS was calculated from the end of SBRT until the last follow-up or the patient’s death.

Moreover, disease-free survival (DFS) was defined as the time between the primary treatment (surgery/radiotherapy) for prostate cancer at diagnosis and the first session of SBRT for oligorecurrent patients. Treatment-related toxicity was assessed using the Common Terminology Criteria for Adverse Events 5.0 (CTCAE 5.0) scale [11].

Descriptive statistics were used to summarize patient’s features. Normality of the distributions was assessed using the Kolmogorov–Smirnov test. Categorical variables were presented as frequencies or percentages and compared with the use of the Chi-Square test or the Fisher’s exact test, as appropriate. Continuous variables were presented as means ± SD (in case of a normal distribution), or medians, IQR and min/max (in case of a skewed distribution) and compared with the use of Student’s t test, Anova, or the Mann–Whitney and Kruskal–Wallis test; correlations among variables by the Pearson’s or Spearman’s rank correlation test.

Logistic regression analyses were performed with the log-rank test and the Kaplan–Meier method to correlate survival with the following variables: 1 or > 1 metastases, sites of metastases (nodes and bone), location of node metastasis (N1 and M1a), primary tumor Gleason Score, D’Amico risk group, PSA values at the onset of metastases (dichotomized at the median value), and PSA doubling time (dichotomized at the median value). A *P* value < 0.05 was considered statistically significant. All statistical calculations were performed using SPSS Software version 20.0 (SPSS Inc, Chicago, IL).

## Results

According to the inclusion criteria of the study, 82 patients and 118 lesions were retrospectively analyzed. In Table 1 baseline patients’ and tumor characteristics are reported.

**Table 1:** Baseline patients and tumor characteristics

Age at PCa diagnosis (yr), median (IQR)	68 (59–72)
PSA at PCa diagnosis (ng/ml), median (IQR)	10 (7–15)
c/pT Stage at diagnosis, n (%)	
T1c	1 (1.2%)
T2	33 (40.2%)
T3	43 (52.4%)
T4	5 (6.1%)
c/p N Stage at diagnosis, n (%)	
N0	76 (92.7%)
N1	6 (7.3%)
Gleason Score sum at diagnosis, n (%)	
6	12 (14.6%)
7	42 (51.2%)
> 7	28 (34.1%)
D’Amico risk group, n (%)	
Very low/low	8 (9.7%)
Intermediate (favorable/unfavorable)	20 (24.4%)
High/very high	54 (65.9%)
Type of primary treatment, n (%)	
RP only	15 (18.3%)
RT only	13 (15.9%)
RP plus RT	54 (65.8%)
RT field, n (%)	
Prostate only ± seminal vesicles	59 (88%)
Whole pelvis RT	8 (12%)
ADT at primary treatment, n (%)	
Yes	29 (35.4%)
No	53 (64.6%)
Interval from diagnosis to oligometastases (mo), median (IQR)	55 (33–92)
Age at recurrence (yr) median (IQR)	72 (65–76)
PSA at recurrence (ng/ml), median (IQR)	1.3 (0.6–2.3)
Metastatic site, n (%)	
Nodal	69 (84.1%)
Bone	13 (15.9%)
Metastatic nodal site, n (%)	
N1	47 (68.1%)
M1a	17 (24.6%)
N1 + M1a	5(7.2%)
No. of lesions treated, n (%)	
1	29 (42%)
> 2	53 (68%)
SBRT schedule (node)	
35 Gy/7fr	19
30 Gy/5fr	3
36 Gy/6fr	17
42 Gy/7fr	1
31 Gy/5fr	1
32 Gy/5fr	1
35 Gy/5fr	3
45 Gy/6fr	3
40 Gy/5fr	9
27 Gy/3fr	2
30 Gy/3fr	3
50 Gy/5fr	1
42 Gy/4fr	1
36 Gy/3fr	3
48 Gy/4fr	5
SBRT schedule (Bone)	
30 Gy/3fr	6
40 Gy/5fr	1
24 Gy/3fr	1
35 Gy/5fr	1
30 Gy/5fr	3
25 Gy/5fr	1

At the time of analysis, the median follow-up was 52 months (range 13–127 months). The DFS between primary tumor treatment and the onset of oligorecurrences was 55 months (range 33–92 months). Most patients (69/82) were affected by nodal oligometastases (84.1%), while the remaining 13 (15.9%) by bone metastases. Forty-two percent of patients were treated for single metastases, while the remaining 58% received the SBRT treatment for two or more metastases. No patient was treated for more than three metastases.

**Fig. 1:**
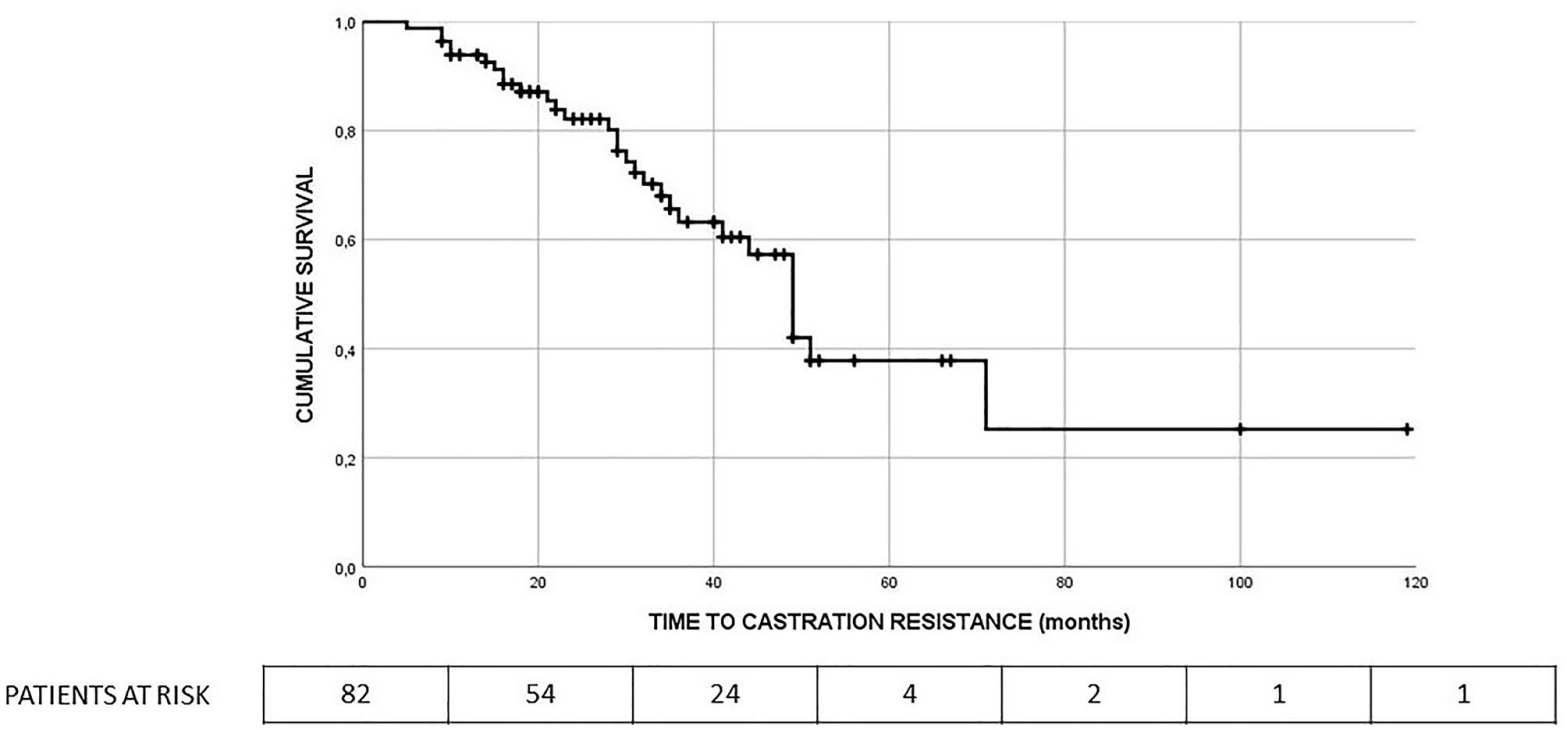
TTCR-free survival curve for the entire population (median 49 months, 95% CI 43.6–54.4 months)

The median TTCR for the entire population of the study was 49 months (95% CI 43.6–54.4 months). The 1- and 2-year TTCR free survival rates were 94% and 82%, respectively. Figure 1 depicts TTCR-free survival curve.

The median ADT-free survival for the entire population of study was 18.2 months (95% CI 14.5–21.9 months). The 1- and 2-year ADT-free survival rates were 66% and 32%, respectively. At the time of analysis, 52 patients were still in the castration-sensitive phase of the disease. In this cohort of patients, the median ADT-free survival was 20 months (range 3–69 months). On the other hand, during follow-up 30 patients switched to the castration-resistant phase of disease. In this last group of patients, the median ADT-free survival was 20 months (range 4–50 months). After the ADT administration, the median castration-sensitive phase was 29 months (range 5–71 months).

Globally, 10-year OS was 96.3%, whereas 5-year LPFS was 90.2%. As far as adverse events are concerned, SBRT was well tolerated, with no gastrointestinal or genitourinary grade ≥ 2 acute/late toxicity.

At statistical analyses, no correlation was found between the variables analyzed and the survival outcomes were analyzed, including the TTCR and ADTFS (Tables 2 and 3).

**Table 2:** Statistical analytical results

Covariate	TTCR HR (95% CI)	p-value
D’Amico risk group, n (%)		
Very low/low	1	
Intermediate	0.58 (0.07–5.17)	0.87
High/very high	0.75 (0.09–5.71)	
Gleason score sum:		
6	1	
7	3.36 (0.49–23.12)	
> 7	5.24 (0.67–41)	0.27
PSA level at time of metastases, ng/ml:		
< 2 ng/ml	1	
> 2 ng/ml	0.68 (0.28–1.64)	0.39
Location of metastasis:		
Node	1	0.825
Bone	0.89 (0.31–2.56)	
Location of node metastasis		
N1	1	0.49
M1a	1.41 (0.53–3.73)	
No. of lesions		0.52
1	1	
> 1	0.75 (0.31–1.80)

**Table 3:** Statistical analytical results

Covariate	ADT FS HR (95% CI)	p-value	ADT FS (median, mo.)	p-value
D’Amico risk group, n (%)		0.342		0.315
Very low/low	1		12.6	
Intermediate	1.29 (0.56–2.94)		13.8	
High/very high	0.86 (0.41–1.82)		18.2	
Gleason score sum				
6	1	0.15	9.7	0.15
7	0.83 (0.43–1.58)		18.5	
> 7	0.55 (0.27–1.1)		18.2	
PSA level at time of metastases, ng/ml				
< 2 ng/ml	1	0.069	18.2	0.065
> 2 ng/ml	1.52 (0.97–2.39)		15.6	
PSA doubling time		0.64		0.917
< 5 months	1.12 (0.69–1.81)		18.2	
> 5 months	1		18.2	
Location of metastasis		0.64		0.64
Node	1		18.2	
Bone	1.15 (0.63–2.1)		13.8	
Location of node metastasis		0.38		0.38
N1	1		18.2	
M1a	0.79 (0.47–1.34)		19.2	
No. of lesions				
1	1	0.74	18.2	0.74
> 1	1.08 (0.68–1.71)		18.2	

## Discussion

The optimal combination of MDT and ADT is still a matter of study. During the last years, several studies have explored whether there could be a significant clinical impact by treating metastatic foci of disease by means of MDT in the scenario of oligorecurrent PC [8, 12].

It is true that most of the data are focused on the castration-sensitive oligorecurrent PC [13, 14]. Transversely, all the available literature data confirm the crucial role of MDT in terms of postponing drug administration as well as ameliorating certain survival endpoints [15]. Concerning the first point, the ADT administration delay is particularly important for clinicians to temporarily avoid ADT-related adverse events [16, 17]. Furthermore, in the recent years, ADT-free survival was also proposed as a surrogate end point of the other major clinical outcomes such as OS or PFS considering the importance of delaying an active but time-defined systemic treatment [7, 8].

On the other hand, it remains field of interest which patients could be the optimal therapeutic sequence: upfront MDT or first-line ADT followed by MDT or both in a concomitant approach. During the St Gallen Advanced Prostate Cancer Consensus Conference [18], a disagreement was registered concerning the palliative ADT administration in the PC oligorecurrent patients. “ADT-believers” would administer first-line ADT to ensure its favorable effect on the natural history of castration-sensitive PC, whereas “MDT-believers” mostly appreciate the role of local ablative therapies (surgery or SBRT). Furthermore, a recent meta-analysis driven by Connor et al. comparing four randomized clinical trials showed that men with oligorecurrent castrate-sensitive PC seem to have an advantage in terms of oncologic outcomes by MDT [19]. A synergistic point of view could be found if it will be demonstrated that using upfront MDT followed or not by ADT does not reduce the castration-sensitive period, especially when ADT is administered after MDT failure.

In this single arm type of study, the TTCR was measured, which was quite impressive with median time of 48 months (assuming time zero is the last day of SBRT). Additionally, the median castration-sensitive phase after ADT administration was 29 months, suggesting that once ADT started, it took a median of 29 months before castration resistance developed. In the absence of randomized trials, a comparison could be conducted with previous historical publications according to which after first-line ADT, disease progression represents a common event due to the development of the castration-resistant phase that generally occurs at a median follow-up of 24–36 months [2–4].

In this setting, it is worth mentioning the results of the STOMP [13] trial which compared MDT versus active surveillance in oligorecurrent PC. In the recent update of the study, authors reported 5-year CRPC-free survival rate of 76% versus 53% for MDT and surveillance arms, respectively. In our series, 5-year CRPC-free survival rate was about 65%, which is reasonably in line with the current literature.

In contrast, the results of the TOAD trial [20] seem to be quite similar to ours in terms of ADTFS. This Australian paper compares delayed or immediate ADT administration for PSA-relapse PC patients and median time to ADT administration for the delayed arm is 18 months, which seems to be comparable to our median ADTFS. Yet, in our series, ADTFS was calculated from the last session of SBRT to the administration of ADT for the polymetastatic disease conversion. If we had to calculate ADTFS starting from the data of the biochemical relapse, our median ADTFS would reach 24 months, which is a reasonable achievement in this subgroup of patients. This scenario highlights the need of a standard definition of ADTFS in oligometastatic patients treated with MDT.

To the best of our knowledge, this is the first study that has demonstrated the synergistic action of sequential MDT and ADT to prolong the castration-sensitive “window”. A possible explanation could be related to the different mechanisms of action by ablative irradiation and ADT. It is recognized that androgen-deprived PC metastasis could further metastasize and a clonal relationship among the different “waves” of PC metastases is reported. Thus, clonal heterogeneity inside each metastasis could be able to bypass ADT control by a selection process of sub-clones. This last phenomenon could lead toward a convergent path of therapeutic resistance. MDT “freezes” this process since the beginning thanks to its ablative effect, which does not depend from clonal ADT sensitivity.

The preliminary identification of the “real” oligorecurrent PC patients still remains therefore crucial before offering SBRT. In this scenario, the newer metabolic imaging remains crucial to overcome the so-called “iceberg theory” [21, 22]. According to this last principle, MDT efficacy could be heavily affected by the limits in identifying the clinical disease diagnosed only using conventional imaging. The adoption of new metabolic tracers could optimize the selection process for MDT-strategy due to the possibility to detect macroscopic foci of disease at low PSA levels. In the present study both Choline and PSMA tracers were used to diagnose oligorecurrent disease, leading to potential biases. Indeed, in a previously published article from Mazzola and colleagues, PET-PSMA-guided MDT seems to offer an advantage in terms of ADT-free survival if compared to PET-choline-guided SBRT in the oligorecurrent PC [23].

The main interest of the current study for future research relies on its sample dimensions, follow-up duration, and its primary outcome (i.e., castration-resistant disease-free survival).

On the other hand, the possible limitations are (i) the retrospective nature of the study that may lead to some critical biases and (ii) different metabolic imaging used at time of oligorecurrence. Finally, last but not least, there was no comparison to another group of patients who did not get SBRT for oligorecurrent PC disease and managed with a more conventional/standard approach of observation and eventual ADT. For this reason, well-designed prospective trials are strongly advocated to validate the present retrospective study.

## Data Availability

Datasets are available upon reasonable request.
